# Influence of hypertension and other risk factors on the onset of sublingual varices

**DOI:** 10.1186/s12903-021-01604-1

**Published:** 2021-05-04

**Authors:** Agostino Accardo, Lorenzo Pascazio, Fulvia Costantinides, Fabio Gorza, Giulia Silveri

**Affiliations:** 1 Department of Engineering and Architecture, University of Trieste, Trieste, Italy; 2 Department of Geriatrics, University of Trieste&ASUGI, Trieste, Italy; 3 Department of Medical, Surgical and Health Sciences, University of Trieste&ASUGI, Trieste, Italy

**Keywords:** Sublingual varices, Blood pressure, Risk factors, Hypertension, Multivariate analysis

## Abstract

**Background:**

Sublingual varices (SV) are dilatations of tortuous veins that increased with age. Previous studies showed that this pathology could be correlated to some risk factors such as hypertension, age, gender and diabetes mellitus. In this study we evaluated, on a large number of subjects, the relationship between SV and different grades of hypertension as well as some risk factors extending the analysis to new risk factors such as dyslipidemia, obesity and antihypertensive therapy, modelling a possible dependence of SV on all these factors.

**Methods:**

In the study 1008 subjects, 284 with and 724 without SV, were examined. The blood pressure was measured in office condition and, to exclude subjects with *white coat syndrome* or *masked hypertension*, also using a 24 h Holter pressure monitor. Hypertensive subjects were divided in resistant, drugs controlled (compensated) and patients with prior unknown hypertension (new diagnosed) groups. The presence or absence of SV as well as of the risk factors was assessed clinically. We tested the influence of age on the presence of SV by using the chi-square test and the relation between each risk factor and SV by the Cochran–Mantel–Haenszel test. Finally, we carried out a multivariate regression tree analysis in order to predict the presence of SV.

**Results:**

We confirmed the influence of age on SV and found a significant relationship between SV and both the compensated and resistant hypertension grades. We highlighted a relationship between SV and dyslipidemia in subjects with new diagnosed hypertension, and between SV and smoking in subjects with compensated hypertension grade. The regression tree showed a classification accuracy of about 75% using as variables hypertension grades, age and antihypertensive treatment.

**Conclusions:**

We confirmed the SV dependence on age, resistant hypertension and smoking, highlighting a new association with dyslipidemia in new diagnosed hypertensive subjects and new relations depending on the hypertension grades. Thus, the SV inspection could be used to suggest a lipidologist as well as a hypertension specialist visit for a pharmacological and pressure check particularly in subjects presenting SV and dyslipidemia. However, further parameters are to be considered to improve the sensitivity of the prognostic tree model.

## Background

Sublingual varices (SV) are vascular dilatations clinically characterized by increased tortuous veins. They may be frequently present along the ventral surface of the tongue or floor of the mouth but they may also occur on the lips. The clinical features of SV are usually multiple, irregular, blue-purple, elevated blebs on the ventral and lateral border of the tongue. The lesions are habitually asymptomatic and frequently encountered during routine clinical examination.

Do Egido Vasconcelos et al. [1], in a study of 30 diabetic patients in which were evaluated thirteen different types of mucosal alterations, reported that tongue varicose veins were the most prevalent, even if the pathogenesis of SV is not fully understood. They speculated that this may be due to a change in the connective tissue or weakening of the venous wall [2] and tend to become more prominent with age [3, 4]. The SV are distributed from the posterior part bilaterally to the apex of tongue and are usually detected after age 40 and the incidence increased with age [5]. Kaplan and Moskona reported in a clinic survey of 298 geriatric patients (94 male and 204 women) that varicosities increased between 50 and 99 years old [6]. A study of 22 elderly vegetarians aged 57–75 years found a much lower incidence of sublingual varicosities than that generally reported in older population [7]. In a sample of 281 subjects over 40 years old, Hedstrom et al. [8] highlighted that the presence and the number of varices increased significantly with ageing. This was in accordance to [5], suggesting that SV may be related to abnormalities in the circulation system commonly occurring in specific disease. In addition, Kleinman [9] affirmed, in a double-blind study of 159 patients with and without pulmonary disease that SV are related to the aging process and, prior to the fifth years old, varices may be an indicator of premature aging. Al-Shayyab et al. [5], in a sample of 391 subjects (13–74 years old, 203 males and 118 females), reported that the SV were more relevant in females than in males that could indicate a hormonal influence on SV. Marcondes et al. [10], studying the Brazilian population, showed that the prevalence in females was about 70% greater than in males and that in individuals younger than 50 years old, the incidence was similar in both gender. Same results were reported by other authors [11] that did not found any significant gender difference.

Beside the studies concerning the influence of age and gender on the presence of SV on the tongue, other authors investigated the relation between SV and other potential risk factors such as hypertension, smoking, diabetes mellitus, cardiovascular disease and denture wearing and to be vegetarians [1, 5, 7, 8, 11, 12]. In particular, some authors [5, 8] founded that SV were significantly associated also with smoking, denture wearing, and cardiovascular disease.

Moreover, some authors [11, 12] found that SV were significantly associated with resistant hypertension and smoking, observing that the prevalence of SV in ex-smokers was higher than that of current smokers and concluding that the smoking cannot be a contributory factor of SV onset [11].

However, till now, the relation between the presence of SV and hypertension in specific grades like compensated and new diagnosed hypertension has not yet been studied. With the aim to better understand the influence of hypertension on SV, in this work we investigated their relationship both in subjects presenting resistant grade, in order to confirm the results of previous studies on a large amount of subjects, and in subjects with the other two grades of hypertension. The known influence of age was also examined and taken in consideration. Moreover, we extend the analysis to the influence of other potential risk factors, some of which not yet analysed in the literature, such as dyslipidemia, obesity and antihypertensive therapy, on the incidence of SV in a large sample of subjects ranging from adult to elderly. Clinical implications of the study concern the possibility of using inspection of varices in order to evaluate the opportunity, in subjects with SV, of a specialist visit to check the blood pressure, the cholesterol level and the consequent drug treatment. Finally, in order to predict the presence of SV, we developed a decision support system based on the multivariate regression tree analysis.

## Methods

### Design and setting

This retrospective study was performed from August 2016 to July 2018 at the Ambulatory of Pathophysiology for Elderly of the Geriatric Department (ASUGI, Trieste, Italy).

### Study population

Subjects were included following these criteria: (1) no clinical or laboratory evidence of secondary arterial hypertension, (2) absence of clinical evidence of hypertension-related complications and (3) no cardiac disease. Moreover, the office Blood Pressure (BP) values must be within a Systolic Blood Pressure (SBP) range of 70–260 mmHg and a Diastolic Blood Pressure (DBP) range of 40–150 mmHg [13]. 1008 consecutive subjects (592 females and 416 males) from 45 to 85 years of age, afferent to the Ambulatory meeting the requirements, have been recruited. The study was carried out according to the principles of Helsinki Declaration. Patients received both verbal and written information about the study and accepted to participate providing written consent. Furthermore, according to the UE Regulation 2016/679 (General Data Protection Regulation) all data were rendered anonymous.

### Procedure

All the subjects were interviewed and the clinical assessment for the presence or absence of SV was carried out by inspection and palpation with the help of the standard set of examinations, like standard lighting and dental mirror [14]. Patient moved the tongue upward and bilaterally so that the sublingual veins could be examined. The SV were classified into two grades depending on either SV absent/few visible (724 subjects) or present in a medium/severe form (284 subjects).

According to current guidelines [13], the blood pressure was at first measured in office condition using a standard mercury sphygmomanometer with a cuff of appropriate size. The BP measurements were taken with the subject in sitting position, after at least 10 min of rest and no vigorous exercise during the preceding 30 min; the average of two consecutive measurements was considered as the BP office value. Based on that value, following the guidelines [13], subject was classified as hypertensive (624, H) (SBP ≥ 140 mmHg and/or DBP ≥ 90 mmHg) or non-hypertensive (384, NH) (SBP < 140 mmHg and DBP < 90 mmHg). In order to exclude from the study subjects presenting ‘white coat syndrome’ or ‘masked hypertension’ syndrome, an ambulatory measurement along the 24-h was carried out by using a Holter Blood Pressure Monitor (Mobil-O-Graph® NG, IEM gmbh Stolberg, Germany), based on oscillometric technique. Subject’s clinical data and drug treatment were collected, physical examination was performed and history of hypertension and other risk factors was carefully controlled. Based on this clinical evaluation and in order to isolate subjects with hypertension who could have an initial organ damage, we divided the hypertensive subjects in resistant hypertensive subjects (198, RH), who were not well controlled by therapy (resistant to treatment, i.e. presenting DBP and SBP values over the hypertension thresholds despite the therapy), compensated hypertensive subjects (413, CH), whose hypertension was fully controlled by drugs therapy (i.e. presenting DBP and SBP values under hypertension thresholds), and subjects with a new diagnosis of hypertension (134, NDH), not known before, not yet treated with drugs and presenting DBP and SBP values over hypertension thresholds. Table 1 shows the distributions of subjects in the four groups distinguishing females and males. In addition to hypertension, the other risk factors examined in this work were age, gender, smoking, obesity, diabetes mellitus, dyslipidemia and antihypertensive therapy. To better examine the relationship between age and SV, the population was divided in five age groups of 10 years each. The smokers were defined as those that have smoked during the last year while the obesity was defined as having a body mass index greater than 30 kg/m^2^. The presence of smoking, diabetes mellitus, obesity, dyslipidemia, antihypertensive therapy was classified as grade 1, the absence as grade 0.

**Table 1:** Distribution of subjects and their age (mean and SD values) with and without SV in the four subject groups

	NH	NDH	CH	RH
*NSV—F*				
# subjects	43	12	72	35
Age (mean ± SD)	69 ± 11	68 ± 9	75 ± 9	73 ± 11
*NSV—M*				
# subjects	14	13	61	34
Age (mean ± SD)	73 ± 10	70 ± 11	72 ± 9	70 ± 9
*SV—F*				
# subjects	125	62	167	76
Age (mean ± SD)	62 ± 12	63 ± 13	68 ± 11	64 ± 11
*SV—M*				
# subjects	81	47	113	53
Age (mean ± SD)	58 ± 12	61 ± 11	67 ± 13	64 ± 11

*NH* non-hypertensive subjects, *NDH* new diagnosed hypertensive subjects, *CH* compensated hypertensive subjects, *RH* resistant hypertensive subjects, *NSV-F* Females without SV, *NSV-M* Males without SV, *SV-F* Females with SV, *SV-M* Males with SV

### Statistical analysis

At first, the relation between the presence of SV and each of the three hypertension grades in comparison with the normotensive condition was evaluated by the chi-squared test. Then the influence of age on the presence of SV was examined considering five age groups (40–50, 50–60, 60–70, 70–80 and 80–90 years old). Since an about linear relation was found for each group, it was approximated by a linear regression and the fitting was evaluated by the R-square statistic and the p-value. Successively, because of the influence of the age represents a confounding variable [3–10, 14], to better estimate the effect of the other risk factors on the presence of SV, all the statistical analyses were performed using Cochran–Mantel–Haenszel test. A p-value of 0.05 was used as the level of statistical significance.

Finally, a multivariate regression tree was applied using as input the risk factors selected by stepwise regression analysis. The very human understandable Classification and Regression Tree (CART) method [15] was used. The method split subjects in two groups, one used in the training phase and the other in the test phase; the sizes of the two groups were respectively 75% and 25% of the total number of data. In the training phase, it builds a tree subdividing, step by step, the subjects presented at the i-th node in two subgroups and selected each time the variable and the threshold to be used for split the subjects in order to minimize the *impurity* of the child nodes. The algorithm begins at the root node in which all subjects are present and it stops when there is only one subject in each node or when the defined maximum number of levels in the tree is reached. Successively, the constructed tree is pruned obtaining a series of sub-trees. The algorithm selects the subtree that minimises a cost function. Successively the tree is tested on the test group of subjects. The algorithm was applied to 1000 different combinations of training and test subject groups, randomly extracted from data, producing 1000 trees.

In order to evaluate the performance of each classifier, the confusion matrix [16] was used to calculate the accuracy, sensitivity, specificity and precision of each tree and we selected that presenting the best ACC. Furthermore, the receiver operating characteristics curve (ROC) was used to depict trade-offs between hit rate (sensitivity) and false alarm rate (1.0-specificity) and the area under the curve (AUC) was calculated [17]. The multivariate regression tree was implemented by using the methods and construction of the MATLAB class *fitctree.*

## Results

Table 2 shows the distribution of subjects with or without SV according to the four subject groups. A significant relation (p < 0.005) between subjects either with compensated or resistant hypertensive grade and the presence of SV was found in comparison with normotensive group. On the contrary, the relation was not significant between new diagnosed hypertension condition and SV.

**Table 2:** Subjects distribution in the four groups and significance level of the difference between NH group and each of the hypertensive groups

		NH	NDH	CH	RH
NSV	# subjects	206	109	280	129
SV	# subjects	57	25	133	69
p-values			n.s	< 0.005	< 0.005

*NH* non-hypertensive subjects, *NDH* new diagnosed hypertensive subjects, *CH* compensated hypertensive subjects, *RH* resistant hypertensive subjects, *NSV* Subjects without SV, *SV* Subjects with SV

**Fig. 1:**
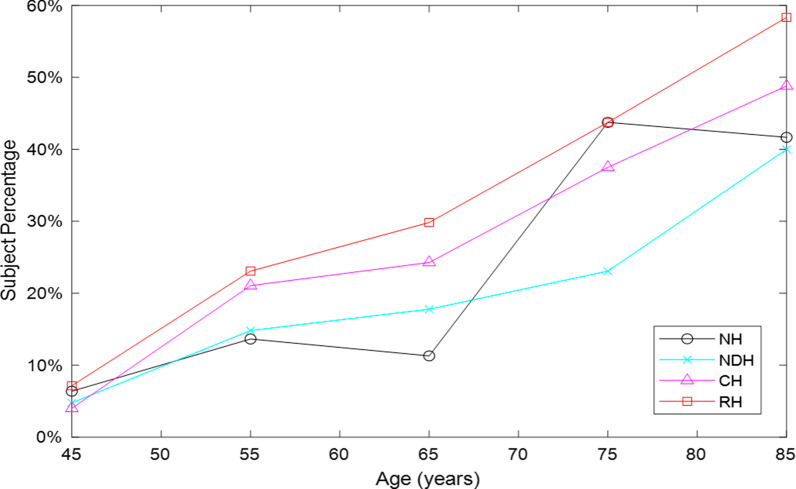
Percentage of the distribution of subjects with SV according to age in the four groups of subjects. *NH* non-hypertensive subjects, *NDH* new diagnosed hypertensive subjects, *CH* compensated hypertensive subjects, *RH* resistant hypertensive subjects

Figure 1 shows the relation between the subject percentage with SV and their age, for all group subjects. The sublingual varices were significantly linearly associated with age in each group with a very high R-square value (Table 3), up to 0.99 (p < 0.001), although NH subjects presented an oscillating behavior among ages. In particular, till 65 years, the percentage of NH subjects affected by SV was lower than 20% followed by a gap between 65 and 75 years till about 50%, persisting at 85 years. On the contrary, in all the hypertensive groups, the percentage of them suffering of SV progressively increased with ageing, presenting different slopes growing from NDH, to CH, till RH group.

**Table 3:** Slopes (m), intercepts (q), R-square (R2) and p-values of the four regression lines fitting the relationship between Age and percentage of subjects affected by SV, showed in Fig. 1

	NH	NDH	CH	RH
m	1.00	0.79	1.06	1.23
q	− 42.1	− 31.1	− 41.8	− 47.6
R^2^	0.79	0.92	0.97	0.99
p-value	0.04	0.01	0.02	0.0006

*NH* non-hypertensive subjects, *NDH* new diagnosed hypertensive subjects, *CH* compensated hypertensive subjects, *RH* resistant hypertensive subjects

Table 4 shows the subject distributions presenting or not SV according to the single risk factors we considered: gender, smoking, diabetes mellitus, dyslipidemia, obesity and antihypertensive therapy, in the four subject groups. The dyslipidemia in NDH group and the smoking in the CH group were the only risk factors presenting a significant relationship with the presence of SV (p-value < 0.05).

**Table 4:** Distribution of subjects with (SV) and without SV (NSV) in the four subject groups according to the six risk factors

	Normotensive subjects		New diagnosed hypertensive		Compensated hypertensive		Resistant hypertensive	
	Gender							
	Female	Male	Female	Male	Female	Male	Female	Male
NSV	125	81	62	47	167	113	76	53
SV	43	14	12	13	72	61	35	34
	Smoking							
	No	Yes	No	Yes	No	Yes	No	Yes
NSV	169	37	89	20	245	35	104	25
SV	51	6	18	7	110	23	55	14
	Diabetes mellitus							
	No	Yes	No	Yes	No	Yes	No	Yes
NSV	195	11	102	7	242	38	105	24
SV	52	5	24	1	109	24	52	17
	Obesity							
	No	Yes	No	Yes	No	Yes	No	Yes
NSV	173	33	82	27	207	73	79	50
SV	44	13	17	8	91	42	48	21
	Antihypertensive therapy							
	No	Yes	No	Yes	No	Yes	No	Yes
NSV	142	64	73	36	1	279	3	126
SV	37	20	16	9	2	131	2	67

**Fig. 2:**
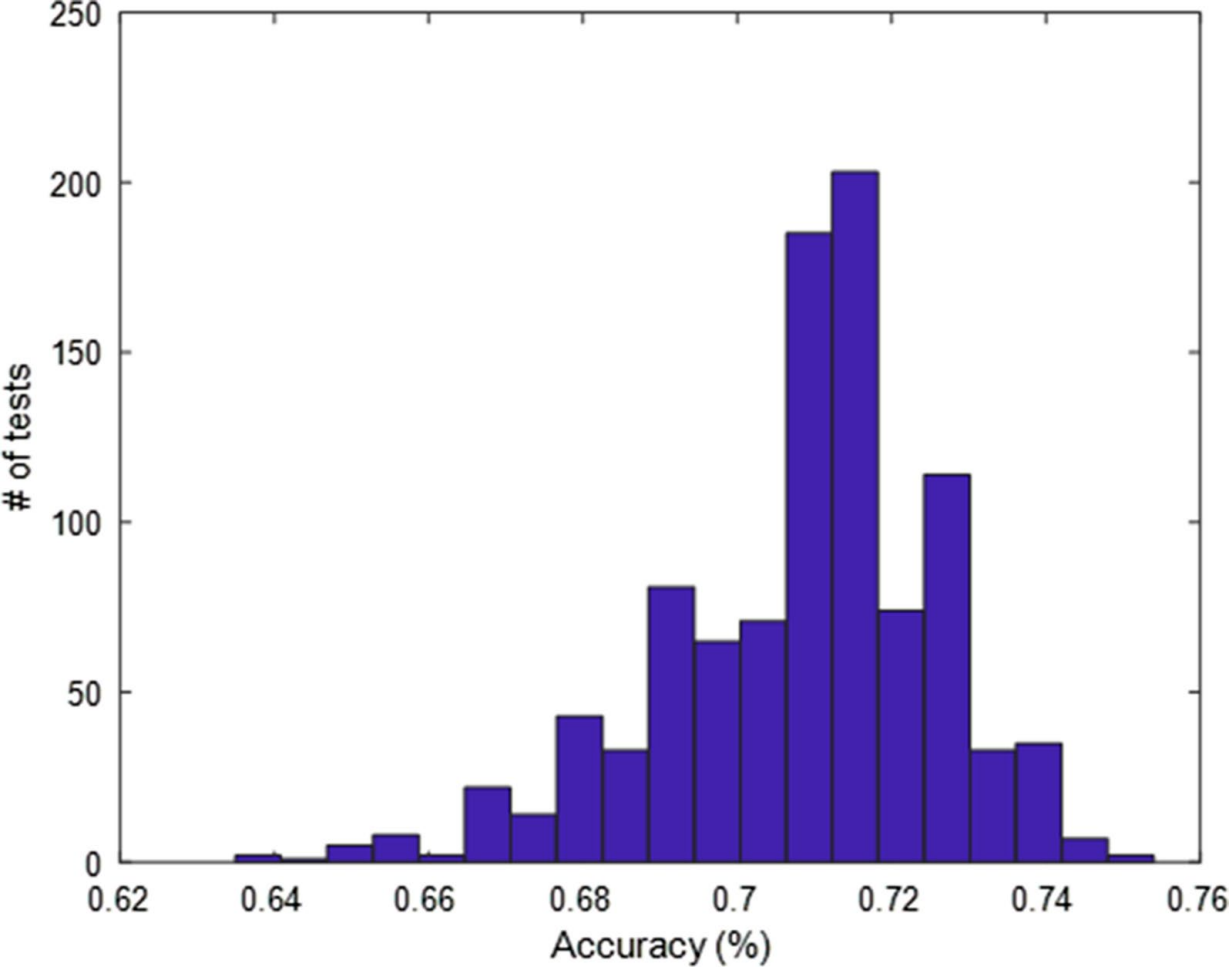
Distribution of accuracy values in the 1000 test repetitions of the multivariate regression tree. Mean value (± 1SD) = 71% ± 2%

**Fig. 3:**
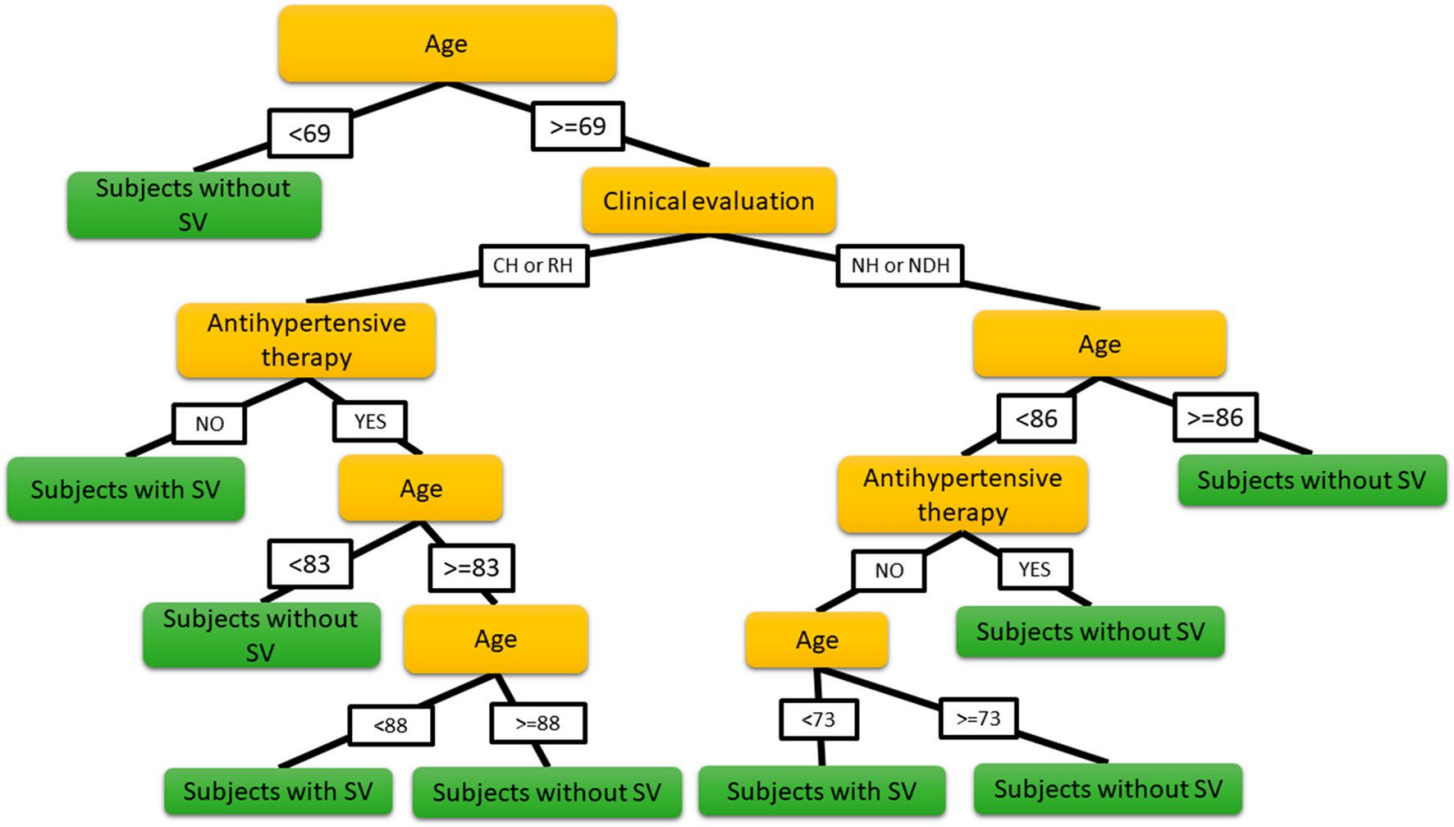
Best classification tree obtained with the CART algorithm. *NH* non-hypertensive subjects, *NDH* new diagnosed hypertensive subjects, *CH* compensated hypertensive subjects, *RH* resistant hypertensive subjects. Each node is the graphical representation of a set of “*if…then*” rules thus, for example, *if* Age is more than 69 years old *and if* clinical evaluation is NH or NDH *and if* Age is more than 86 years old *then* the subject is classified as subject without SV

Stepwise regression analysis between all risk factors highlighted three significant factors (p < 0.05) related to the presence of SV: age, pressure clinical condition (NH, NDH, CH, RH) and presence of antihypertensive treatment. By using these three parameters as input of a multivariate regression tree and repeating the optimization procedure by considering 1000 possible random combinations of input dataset, an accuracy of 75% was reached. In particular, Fig. 2 shows the distribution of accuracy along the repetition tests and the Fig. 3 displays the classification tree presenting the best accuracy. The Sensitivity, Specificity and Precision associated to the presence of SV was of 25%, 97% and 67%, respectively and the ROC analysis highlighted an AUC of 67%.

## Discussion

The present study investigates a large number of risk factors influencing the development of sublingual varices in a very large sample. Until now, only few studies focused the attention on the relation between SV and risk factors such as gender, age, diabetes mellitus, smoking and hypertension in a large population [11, 12], without distinguish the different grade of hypertension. Thus, in our work, beside normotensive subjects, we examined three different hypertension situations, such as new diagnosed, resistant and compensated, clinically identified, excluding subjects presenting either masked hypertension or white coat syndrome [18–21] that could alter the blood pressure evaluation by using the ambulatory measurement along the 24-h.

Figure 1 shows a significant linear increase of with age in all the four subject groups exhibiting highest occurrence in the range between 70 and 85 years old, confirming the results of [4–6, 8] found on hypertensive patients without distinguishing the clinical grade, suggesting that the arising of SV could be compounded by the aging process. This could be due to the changes in the morphology of connective tissue and blood vessels with age so that SV could be mainly attributed to abnormalities in the circulatory system more present in elderly. However, our findings demonstrate that in the not well controlled hypertensive condition the percentage of subjects presenting SV increases with ageing more than in well controlled hypertension and that in the new diagnosed subjects the increment is less marked than in the other two situations. Therefore, for similar ages, a resistant hypertensive subject is more likely to develop SV than a compensated or a new diagnosed one. A possible explanation is that patients with pharmacologically poorly controlled hypertension show more vascular damage and greater cardiovascular events and that lower levels of vascular disease are present in patients with optimal drug treatment on hypertension and in patients with new diagnosed hypertension [22]. Moreover, normotensive subjects presented a particular peak value in the age group between 70 and 80 years old, possibly due to the presence of some risk factors and to the particular extracted sample.

In addition, the results (Table 2) underlined a relation between SV and either well controlled or resistant hypertension, the latter according to the results of [11, 12] but in contrast with the results of Bhaskar et al. [4], that did not reveal a correlation between SV and 384 subjects presenting resistant hypertension, suggesting that a possible explanation was due to circulatory anastomosis in the venous system of the tongue [4]. The relationship between SV and both compensated and not well controlled hypertension could be related to the presence of hypertension for a greater number of years than in new diagnosed hypertension, producing in both cases vascular damages.

Concerning the relationship between SV and gender, our findings showed (Table 4) that we did not find a significant relation between gender and SV confirming the results of [8, 11], but in contrast with [5] that underlined that female were more likely to have SV, suggesting an hormonal influence on SV. In contrast to the results of another study [8], which found an increase to 58% of SV presence in smokers without hypertension, our results suggested that there was no significant association between smoking and SV in normotensive subjects. This maybe because of the smoking has a preventive effect on the development of varicose veins due to vasoconstrictive effect of nicotine, as suggested by [5, 11]. Analyzing hypertensive subjects, Hedstrom et al. [12] underlined an association between the prevalence of SV and non-smokers with hypertension of 44.7% and of SV and smokers with hypertension of 64.7%; we partially confirmed this result founding a significant relationship between smokers and the presence of SV, only for CH group, with lower percentage values (40% and 31%, respectively).

We obtained a significant relationship between SV and dyslipidemia only in subjects with new diagnosed hypertension not yet reported in the literature. However, the relationship seems to be present, albeit non-significantly, even in long-lasting hypertensive patients (compensated), although a greater number of subjects is required to verify this link. Hypertension rarely occurs on its own and often clusters with other cardiovascular risk factors such as dyslipidemia and glucose intolerance producing a multiplicative risk effect. A clinical implication of this result suggests the use of a simple inspection of varices in subjects presenting dyslipidemia in order to evaluate the suitability of a specialist visit for a check on the presence of hypertension and, if present, its correct pharmacological control.

Furthermore, no association between SV and diabetes mellitus was found, even if [1] underlined that in diabetic subject the most frequent abnormalities were lingual varicosity and that the prevalence of oral abnormalities may be the reflection of the different physiological behaviors of the two clinical types of diabetes. Our results could be justified by the limited number of subjects presenting diabetes. Finally, for the first time in the literature, we found that obesity and antihypertensive therapy showed no relation with the presence of SV, probably a better stratification of obesity would be appropriate.

In order to identify subjects with and without SV, we applied a machine-learning algorithm based on the CART method. By using stepwise regression, only three parameters were identified as input of the algorithm: age, pressure clinical condition and presence of antihypertensive therapy. The resulting tree classifiers (Fig. 3) presented a high SV specificity, i.e. a high probability to predict correctly the absence of SV, a low sensitivity, i.e. a low ability to predict correctly the presence of SV as well as good accuracy (75%) and AUC (67%). The proposed technique demonstrates the powerful capability of some risk factors and of CART technique in differentiating subjects with and without SV. However, despite a good accuracy, the parameters considered are not sufficient to predict with high sensitivity subjects with SV, thus it will be necessary to identify other factors to take into consideration such as hypertension-mediated organ damage indexes, extracted from ECG, echocardiography, echo color-doppler of carotid, CT or MR brain examination.

## Conclusion

In conclusion, the analysis carried out in this work in a sample of 1008 subjects confirmed the SV association with age, pointing out SV differences among subjects presenting different hypertension grades*.* Thus, inspection of varices could be a simple and inexpensive method to send the patient to the hypertension specialist, the lipidologist or simply to the general practitioner for a check on the presence of hypertension and, if present, its correct pharmacological control. If examined in dentistry it could be referred to hypertensive specialists. Moreover, the results confirmed a significant association between the presence of SV and subjects with resistant hypertension highlighting a further relation also with subjects presenting compensated hypertension. Since about 40% of population are unaware of being hypertensive or do not have a good pharmacological hypertension control, this may be relevant because discovering a subject with varicose veins would mean re-evaluating him for blood pressure. About some other risk factors, new relations between dyslipidemia and the presence of SV, only in new diagnosed hypertension, and between smoking and SV in compensated hypertensive subjects, were found. Therefore, the presence of SV could lead to a re-evaluation of the patient for all cardiovascular risk factors. Although some studies had underlined the relationship between gender and diabetes and SV [8, 11, 12], these risk factors showed no influence on the presence of SV, probably due to a wide subjects heterogeneity in our sample.

Finally, the combination of some risk factors, selected by stepwise regression, and of a CART algorithm demonstrated to be a possible tool for a prognostic indicator of the presence of sublingual varices, obtaining an accuracy of 75%. However, further studies are need to identify other factors able to increase the sensitivity and accuracy values obtained from the current tree classifier also applying other machine learning algorithms as support vector machines or extreme learning machines.

## Data Availability

The datasets generated and/or analyzed during the current study are not publicly available due the policy of ASUGI Hospital but are available from the corresponding author on reasonable request.

## Abbreviations

SV: Sublingual varices
BP: Blood pressure
SBP: Systolic blood pressure
DBP: Diastolic blood pressure
CH: Compensated hypertensive subjects
NDH: New Diagnosed of hypertensive subjects
H: Hypertensive subjects
NH: Non hypertensive subjects
CART: Classification and regression tree method
ROC: Receiver operating characteristics curve
AUC: Area under the curve
